# Metadynamic metainference: Enhanced sampling of the metainference ensemble using metadynamics

**DOI:** 10.1038/srep31232

**Published:** 2016-08-26

**Authors:** Massimiliano Bonomi, Carlo Camilloni, Michele Vendruscolo

**Affiliations:** 1 Department of Chemistry, University of Cambridge, Lensfield Road, Cambridge CB2 1EW, UK; 2 Department of Chemistry and Institute for Advanced Study, Technische Universität München, Lichtenbergstrasse 4, D-85747 Garching, Germany

## Abstract

Accurate and precise structural ensembles of proteins and macromolecular complexes can be obtained with metainference, a recently proposed Bayesian inference method that integrates experimental information with prior knowledge and deals with all sources of errors in the data as well as with sample heterogeneity. The study of complex macromolecular systems, however, requires an extensive conformational sampling, which represents a separate challenge. To address such challenge and to exhaustively and efficiently generate structural ensembles we combine metainference with metadynamics and illustrate its application to the calculation of the free energy landscape of the alanine dipeptide.

Effective descriptions of complex systems are achieved when a variety of sources of information, including experimental measurements and theoretical models, are combined. Several challenges, however, need to be addressed to obtain accurate and precise models^1^. First, both random and systematic experimental errors, whose level varies depending on the technique used, should be taken into account to properly weigh each element of information in the modelling. Second, one should consider that the prediction of an experimental observable from a model, which is used to quantify the fit of a given model to the observed data, may be inaccurate. Third, systems in equilibrium conditions populate multiple structural states, so that experimental measurements often probe the entire ensemble, rather than individual conformations.

Recently, we have introduced metainference^2^, a Bayesian inference method that addresses all the challenges described above and enables modelling conformational ensembles by properly integrating prior information with noisy experimental data. This approach extends inferential structural determination^3^, in which the level of noise of experimental data is inferred along with structural models, to heterogeneous systems and ensemble-averaged data. In metainference, multiple replicas of the system are modelled in parallel so that experimental observables predicted from the models and calculated as averages over the replicas are compared to experimental measurements, given the unknown level of noise in the data. Notably, metainference reduces to the maximum entropy replica-averaged modelling^4^,^5^ in the limit of low data noise and to standard inferential structural determination^3^ when experimental data are not ensemble averages.

While metainference provides in principle a rigourous way to obtain ensembles of models consistent with the available experimental data, the actual generation of such models remains a computationally demanding task. Traditional methods, including molecular dynamics (MD) and Monte Carlo (MC) simulations, are often inadequate to explore, in an affordable computational time, complex free energy landscapes in which relevant states are separated by high free-energy barriers. To accelerate sampling, metadynamics^6^ (MetaD) has been proved to be particularly effective^7^, also in combination with replica-averaged modelling^8^,^9^,^10^ (RAM). MetaD is based on the introduction of a time-dependent bias potential on selected descriptors of the system, or collective variables (CVs), that ideally should include all those degrees of freedom that are difficult to sample in an affordable computational time. The MetaD bias potential accellerates sampling by discouraging visiting regions of the CV space previously explored and provides an estimate of the free energy as a function of the selected CVs. The choice of a limited set of descriptors to capture all the slow modes of a system has always been proved to be a challenging task. In this context, the recently introduced parallel bias metadynamics^11^ (PBMetaD) attenuates this problem by simultaneuosly applying multiple low-dimensional bias potentials, rather than exploring the multidimensional space of all CVs. In this way, a larger number of CVs can be used and the probability of missing slow degrees of freedom is reduced. Furthermore, the low-dimensional free energies as a function of the individual CVs can be calculated directly from the bias potentials and the full high-dimensional free energy can be easily recovered by standard reweighting techniques^12^.

Here we present metadynamic metainference (M&M), an approach that combines the ability of metainference to model heterogeneous systems by integrating noisy experimental data and prior knowledge of a system, with the enhanced sampling provided by PBMetaD. We introduce the M&M theory and show its application to the prototypical case of alanine dipeptide in vacuo. This system has become a well-established benchmark for many computational techniques, as it is characterized by multiple structural states that are significantly populated at room temperature and separated by high free-energy barriers.

## Results

### Metainference

First, we summarise the theory of metainference^2^. This approach quantifies the extent to which a distribution of models based on prior knowledge of the system is modified by the introduction of *N*_*d*_ experimental data points **D **= [*d*_*i*_], which are subject to random and systematic errors and averaged over the entire distribution. The model is defined by the conformational state *X* of the system and by other parameters, such as the level of noise in the data *σ*. In the spirit of the replica-averaged modelling based on the maximum entropy principle^4^,^5^, we consider a finite sample of the distribution of models by simulating a set of *N* copies (or replicas) of the model. The generation of models is then guided by a score, or energy function, defined by the negative logarithm of the metainference *posterior probability E*_*MI*_ = −*k*_*B*_*T* log*p*, where *k*_*B*_ is the Boltzmann constant, *T* the temperature of the system and


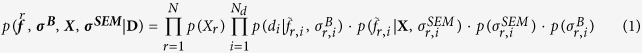


where 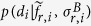 is the conditional probability of data *d*_*i*_ given 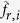 and the uncertainty parameter 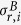, 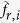 is the average of the function *f*_*i*_ used to predict the experimental observable *i* from a model (*forward model*) calculated on an infinite number of replicas, and 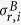 describes random and systematic errors in the experimental data as well as errors in the forward model. 
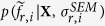
 is the conditional probability of observing 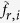 given that the average of *f*_*i*_ is calculated on a finite number of replicas *N*, 
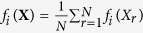
. According to the central limit theorem, 
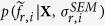
 is a Gaussian distribution and 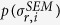 encodes the scaling of the standard error of the mean 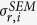 with *N*: 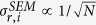. Finally, 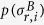 and *p*(*X*_*r*_) are the priors on the uncertainty parameter 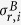 and the state *X*_*r*_, respectively. A derivation of Eq. 1 in the case of a single data point is presented in the Methods section.

### Metadynamics

Next, we review the theory of PBMetaD^11^. In this approach, the sampling is accelerated by the introduction of a time-dependent bias potential *V*_*PB*_ acting on selected CVs, which are functions *S* of the coordinates *X* of the system 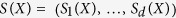



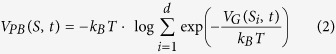


At variance with standard MetaD, in which a single bias potential acts in the multidimensional space of all CVs, here multiple low-dimensional bias potentials are simultaneuosly applied so that the high barriers that characterize multidimensional free-energy profiles can be crossed in a computationally efficient way. The individual potentials *V*_*G*_(*S*_*i*_, *t*) are adaptively built during the simulation by depositing Gaussian functions along the system trajectory in the CVs space, as in standard well-tempered MetaD^13^ (WTMetaD)


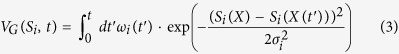


where *σ*_*i*_ is the Gaussian width of the *i-*th CV and *ω*_*i*_(*t*) is a time-dependent energy rate. However, each *ω*_*i*_(*t*) decreases during the simulation according to a scaling recipe different from WTMetaD


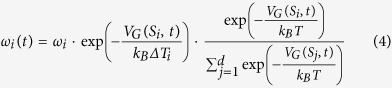


where *ω*_*i*_ is the initial energy rate, and *ΔT*_*i*_ is an input parameter with the dimension of a temperature, which can be used to tune the extent of free-energy exploration. In the long-time limit, each bias potential *V*_*G*_(*S*_*i*_, *t*) converges to the free energy *F*(*S*_*i*_), as in WTMetaD


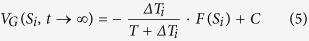


where *C* is an irrelevant constant. Finally, as in the long-time limit *V*_*PB*_ becomes quasi-static, the full high-dimensional free energy can be easily recovered by applying the standard umbrella-sampling reweighting technique^12^.

### Metadynamic Metainference

**Figure 1:**
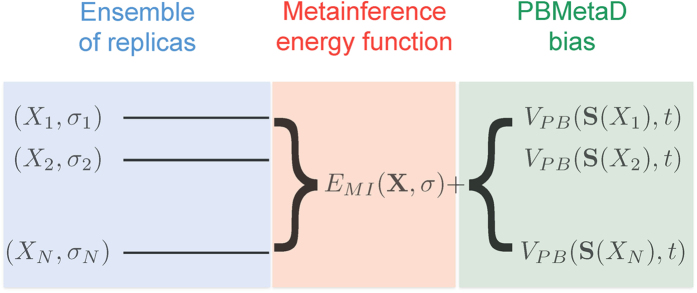
Schematic illustration of the M&M algorithm. In M&M, an ensemble of models is simulated in parallel. Typically a model is composed by the following variables: the coordinates of the system *X* and a list of variables *σ* that represent the level of noise in the data. In principle one can associate a *σ* variable to each experimental data point (Eq. 19), or use an outlier model of noise that requires a single *σ* for all data points (Eq. 20). The replicas are coupled by the metainference energy function, which is composed by different terms: one that describes prior information on the system *X* (for example a molecular mechanics force field), one that describes prior information on the *σ* variable (typically a Jeffrey’s prior), and one term that describes the agreement of the models with the experimental data. This last energy term couples the multiple replicas, as experimental data are expected to be generated by the average over an ensemble of conformations. To accelerate sampling, M&M adds to each replica the bias potential of PBMetaD. This is defined as a function of multiple CVs, which are selected from the user based on *a priori* knowledge of the system to include all the slow modes that need to be accelerated. The PBMetaD bias potential is defined in terms of multiple potentials (one for each CVs), which are stored on a common grid and shared among all replicas. In doing so, the multiple replicas all contribute to fill in parallel the underlying free-energy landascapes and thus to accelerate sampling of the entire ensemble.

We now present the basic theory of M&M. In this combined approach (Fig. 1), an ensemble of replicas of the system is simulated using the metainference energy function. This strategy can be conveniently carried out using a Gibbs sampling scheme, in which the coordinates of the system are sampled by MD and the uncertainty parameters by MC. Additionally, each replica performs a PBMetaD simulation in the same set of CVs. The total M&M energy function is thus


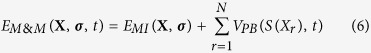


the fact that all replicas bias the same set of CVs using PBMetaD has two important implications. First, replicas can share the low-dimensional bias potentials accumulated during the simulation, as in the multiple-walkers technique^14^. In this way, the benefit of using a high number of replicas is two-fold: the accuracy of the forward model average calculated on-the-fly increases and sampling efficiency scales linearly with the number of replicas. Second, and also as a consequence of the fact that the bias potential *V*_*PB*_ becomes quasi-static in time, the average of the forward model can be easily calculated in the unbiased ensemble as^12^


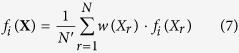


with 
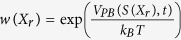
 and 
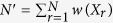
. It should be noted that unbiasing is not strictly necessary, as the incorporation of experimental data into the modelling will correct for the fact that averages are calculated in the biased ensemble. However, more data points are needed to achieve a given accuracy of the modelled ensemble compared to unbiasing using Eq. 7, since in general the ensemble generated by prior information plus PBMetaD potential is more distant from the correct ensemble than that obtained with prior information alone.

M&M differs from RAM^8^,^9^ in two aspects. First, RAM applies a harmonic restraint to keep the average of each predicted observable over the ensemble close to the experimental measurement. The intensity of the restraint is constant and must be chosen as strong as possible, following the maximum entropy principle. In this approach, random and systematic errors cannot be easily incorporated^15^. Second, in RAM replica-averaging is typically coupled with bias exchange metadynamics^9^,^16^ (BEM), so that each replica biases a different CV and random exchanges between replicas are attempted to improve ergodicity. This aspect makes the on-the-fly calculation of unbiased averages over the ensemble a challenging task, and in fact averages are traditionally calculated in the biased ensemble, with each replica experiencing a different bias potential. However, as mentioned above, unbiasing is not strictly necessary when calculating ensemble averages.

### Illustrative example

**Figure 2:**
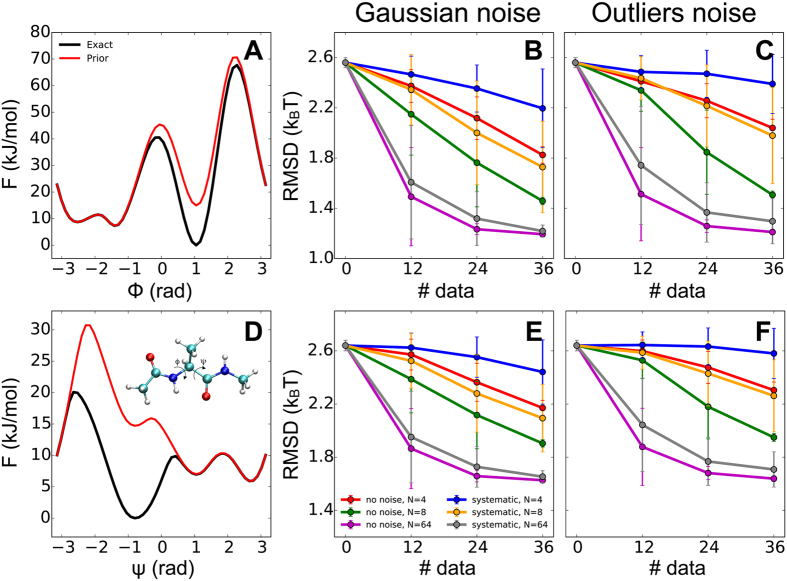
Benchmark of M&M accuracy on the alanine dipeptide in vacuo. We assumed that the ensemble generated by the prior alone (AMBER99SB-ILDN force field, (**A**,**D**) red lines) is inaccurate, and that in the actual ensemble the local minimum *C*_ax_ is more populated (**A**,**D**, black lines). We then used M&M to combine the inaccurate prior with synthetic experimental data generated as averages on the correct ensemble. We calculated the error in the reconstructed free energies as a function of the backbone dihedrals *ϕ* (upper panels) and *ψ* (lower panels), using Gaussian (**B**,**E**) and outliers noise models (**C**,**F**). For both noise models, we benchmarked the method as a function of the number of data points, the level of noise in the data, and the number of replicas used.

To benchmark the accuracy of M&M, we used alanine dipeptide in vacuo (CH_3_-CO-NH-C_α_HCH_3_-CO-NH-CH_3_), which has become a standard test case for many computational techniques^17^,^18^,^19^,^20^,^21^. The most common descriptors for this system are the two backbone dihedral angles *ϕ* and *ψ*. The free energy in the Ramachandran^22^ map 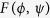 is characterized by two main minima, known as *C*_7eq_ and *C*_ax_, which are separated by a high free-energy barrier and connected by three different low free-energy paths^17^. We described this system using as prior information the AMBER99SB-ILDN force field^23^, which results in a free energy difference and barrier between *C*_7eq_ and *C*_ax_ in the monodimensional projection 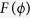 of −7.5 kJ/mol and 38 kJ/mol, respectively (Fig. 2A, red line). We assumed that this prior is inaccurate and that in the actual distribution (Fig. 2A, black lines, and SI) the free energy of the local minimum *C*_ax_ is 15 kJ/mol lower than AMBER99SB-ILDN. The goal is to evaluate the accuracy of the ensemble reconstructed by M&M using the inaccurate prior and (synthetic) experimental data generated by calculating averages in the correct ensemble (SI). As data we used the distances between all the (non-bonded) heavy atoms of the dipeptide, resulting in 36 data points (Table S1, third column). Only a few of these data points can discriminate fairly well between *C*_7eq_ and *C*_ax_ (Table S1, fourth and fifth columns) and thus they are sensitive to a change in population of these two states and can be used to improve the accuracy of the ensemble obtained by the prior information alone.

In the M&M scheme, we used the four dihedrals *ϕ*, *ψ*, *θ*, and *ζ* as PBMetaD CVs, with Gaussian widths and bias factors 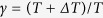 equal to 0.35 rad and 8 for all CVs, respectively, initial Gaussian height of 1.2 kJ/mol, and deposition stride of 1 ps. We define *accuracy of an ensemble* the root mean square deviations (RMSDs) of the free-energy estimates along *ϕ* and *ψ* obtained from the PBMetaD bias potentials (Eq. 5) from the reference ones (Fig. 2AD, black lines). We extensively assessed the convergence of our simulations by monitoring the diffusion in the CV space under the effect of the PBMetaD bias (Fig. S1) and evaluating the RMSDs as a function of simulation time (Fig. S2). The reported accuracies are obtained from the free energy estimates at the end of the M&M runs, averaged over 100 independent simulations. Averages of the forward models were calculated using the reweighting formula of Eq. 7 and replicas shared the bias potentials following the multiple-walkers scheme. The total simulation time for each run was 120 ns. We determined the accuracy of our approach as a function of the number of data points, the level of noise in the data, and the number of replicas. We tested two different noise models: a Gaussian distribution with one uncertainty parameter per data point (Eq. 19) and the outliers model with one typical uncertainty per dataset (Eq. 20). In both cases, we used an uninformative Jeffrey’s function 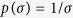 to model the uncertainty priors and we fixed the standard error of the mean *σ*^*SEM*^ at 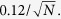 To assess the sampling efficiency and ensemble accuracy provided by PBMetaD, we run a subset of the simulations of the benchmark using the pure metainference approach and coupling metainference with BEM^9^,^16^, in the same way as MaxEnt replica-averaging was combined with BEM in RAM^9^. All simulations were carried out with GROMACS^24^ equipped with the PLUMED plugin^25^. Additional details of the data generation, setup of simulations, and analysis can be found in SI.

**Figure 3:**
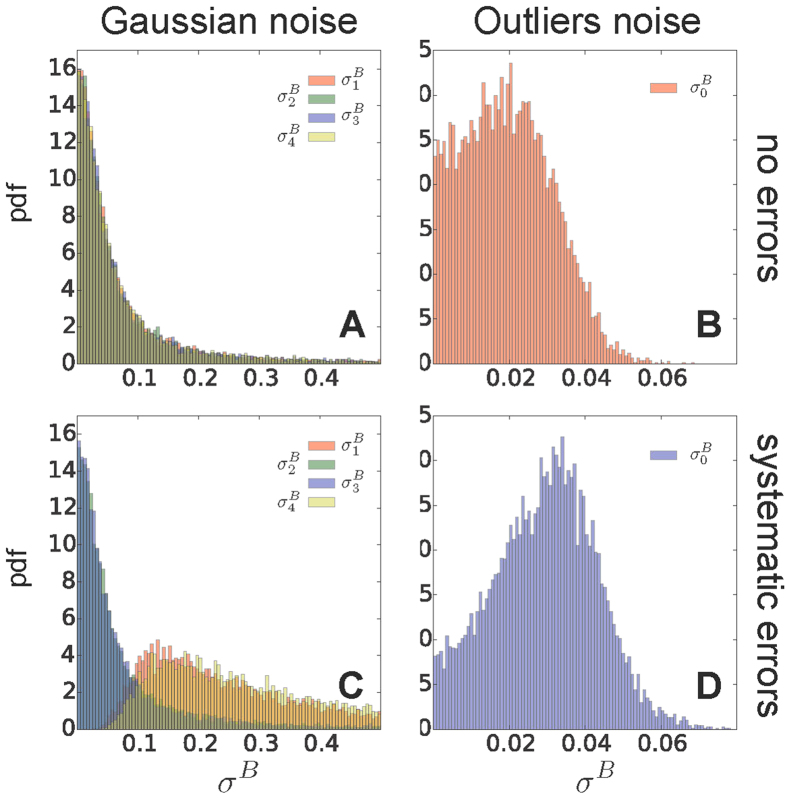
Analysis of the uncertainties inferred by M&M. The distributions of the *σ*^*B*^ parameter in representative runs with 8 replicas and all the 36 data points available, using the Gaussian (AC) and outliers noise models (BD), in absence (top panels) and presence of systematic errors (bottom panels). When using Gaussian noise, we plot the distributions of the uncertainty parameter associated to four representative data points (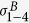). When systematic errors are present, we selected two outliers (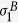 and 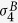) and two data points not affected by error (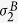 and 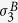). When using the outliers model, we plot the distribution of the typical dataset uncertainty 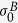.

The accuracy of the M&M ensemble generated using the Gaussian noise model (Fig. 2B,E) increased upon adding new experimental data, both in absence (red, green, and magenta lines) and presence of systematic errors (blue, orange, and grey lines), which affected 20% of data points (SI). It must be noted that a small random error in calculating the average experimental data was still present, since these were calculated from a MD simulation of finite length. This trend was determined by the fact that M&M was capable of automatically detecting the correct level of noise of each data point, as shown by the distributions of inferred uncertainties (Fig. 3A,C), so that noisy points were downweighted in the model construction. As a consequence, a dataset containing outliers was equivalent to a smaller dataset in which errors were absent. Therefore, while at fixed number of data points the accuracy of the ensembles generated using experimental data with no errors was slightly higher, the derivative of the accuracy with respect to the number of data points was similar in presence and absence of errors. In both cases, we also found that the error in the reconstructed free energies along *ϕ* (Fig. 2B) and *ψ* (Fig. 2E) decreases with the number of replicas used, as observed in simpler heterogeneous model systems^2^. The behavior with the number of replicas was also expected, as the accuracy in the calculation of average quantities increases with the dimension of the simulated ensemble. As discussed above, the use of a large number of replicas leads also to an increase in sampling efficiency, as demonstrated for standard MetaD in the multiple-walkers scheme^14^.

In all cases studied, the absolute value of the error depends on the accuracy of the prior, as discussed in detail previously for the heterogeneous model system^2^, and the information content of the data used in the modelling. As a matter of fact, for such small systems as alanine dipeptide, it is quite challenging to design a large number of synthetic experimental data that can discriminate between the two local minima so that their averages in the exact and prior ensembles are markedly different (Table S1). This kind of data would carry the most useful information to correct for prior inaccuracies and reduce the overall error of the modelled ensemble.

The outliers model showed the same trend in accuracy as a function of data points as the Gaussian model (Fig. 2C,F). When no systematic errors were present in the data, the outliers and Gaussian noise model resulted in ensembles of similar accuracy, especially when using 64 replicas and 36 data points, in which case the difference in accuracy between the two models was smaller than 0.02 k_B_T. The distribution of the typical uncertainty of the dataset used in the outliers model (Fig. 3B) was less broad than those of the individual uncertainties in the Gaussian model (Fig. 3A), but it was peaked at the same value.

In presence of systematic errors in the data, the outliers noise model generated ensembles slightly less accurate then those obtained with the Gaussian noise model. In the case of 64 replicas and 36 data points, the accuracy of the outliers noise model was worse than the Gaussian noise model of the order of 0.06 k_B_T. This difference is due to the greater flexibility of the Gaussian noise model, where one uncertainty parameter is assigned to each data point, while in the outliers model uncertainties are marginalized using a unimodal distribution peaked around a typical (unknown) dataset uncertainty. However, the outliers model has the computational advantage of having a single uncertainty parameter to sample. The accuracy of a simpler Gaussian model with one uncertainty parameter for all data points, and thus the same number of parameters of the outliers model, was significantly worse in presence of systematic errors (Figs S3 and S4). Finally, the typical uncertainty inferred using the outliers model (Fig. 3D) was somewhere in between the uncertainties of the Gaussian model for points with and without systematic errors (Fig. 3C).

The benchmark of the pure metainference approach revealed that the metainference simulations of alanine dipeptide in vacuo were not ergodic in the time scale used here. As expected, in the absence of the PBMetaD bias potential the system was not fully capable of sampling exhaustively the entire CV space. In 612 out of 2400 metainference runs, one of the two relevant minima was never visited during the entire course of the simulation, as the high free-energy barrier that separates the two local minima could not be crossed in the limited time scale available (Fig. S5). In the remaining runs replicas were trapped in the initial basin and the results were thus influenced by the initial distribution of replicas (Fig. S6). This behavior is strikingly different from the one observed in the M&M simulations (Fig. S1).

The benchmark of metainference combined with BEM revealed that PBMetaD generates more accurate ensembles and guarantees more efficient sampling. We performed a representative set of simulations from our M&M benchmark on alanine dipeptide *in vacuo*, using BEM as sampling engine. We used the Gaussian noise model with one uncertainty parameter per data point, no systematic errors in the data, 36 data points and 8 replicas. BEM was carried out with the same parameters (Gaussian height, sigma, pace and biasfactor) as in PBMetaD. We used the 4 dihedral angles as CVs, with each replica biasing only one CV. Given the fact that we utlized 8 replicas and 4 CVs, each CV was biased by two different replicas. Exchanges between replicas were attempted every 1000 MD steps. The errors in the free energy estimates along the dihedrals *ϕ* and *ψ* averaged over 1000 simulations were 1.66 ± 0.06 *k*_*B*_*T* and 1.92 ± 0.06 *k*_*B*_*T* for BEM, 1.46 ± 0.03 *k*_*B*_*T* and 1.90 ± 0.03 *k*_*B*_*T* for PBMetaD (Fig. S8). The reason why PBMetaD simulations are more accurate than BEM simulations is that in PBMetaD the averages of experimental observables are calculated in the unbiased ensemble (*i.e.* after removal of the effects of the PBMetaD bias potential) by on-the-fly reweighting (Eq. 7). In BEM this reweighting is not possible, and averages are calculated in the biased ensemble, as the system was simulated using an effective prior information equal to the BEM bias potential plus the molecular mechanics force field. The results of our control simulations show that this effective prior is of inferior quality compared to the one used in PBMetaD simulations, *i.e.* the molecular mechanics force field alone. As a consequence, when using BEM as sampling engine, a larger amount of experimental information is needed to achieve the same ensemble accuracy obtained with PBMetaD. Furthermore, the analysis of the error convergence as a function of simulation time (Fig. S8) demonstrated that PBMetaD is a more efficient sampling engine than BEM when combined with metainference, as shown in Ref. 11 for PBMetaD alone. This is due to the fact that in PBMetaD all the replicas bias the same set of CVs and share the accumulated bias potential.

**Figure 4:**
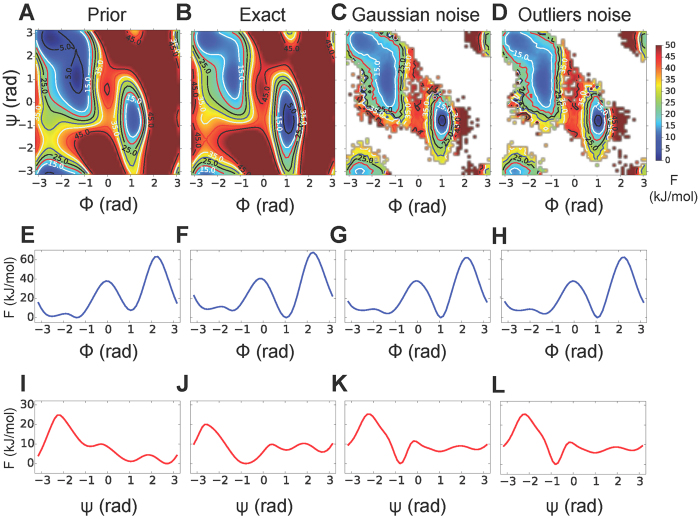
Reweighting of M&M simulations. Free energy of alanine dipeptide as a function of the backbone dihedrals obtained with the AMBER99SB-ILDN prior alone (**A**) and with the correction to lower the free energy of the local minimum *C*_ax_ (**B**). The latter is considered our reference (exact) free energy. Free energy obtained from reweighting a M&M simulation carried out using 64 replicas, all the 36 data points available (without addition of systematic errors), the Gaussian noise model with one uncertainty parameter for each data point (**C**) and the outliers noise model with one parameter per dataset (**D**). The visualization is truncated at 50 kJ/mol from the global minimum. For each case (prior, exact, Gaussian and outliers noise), we also reported the monodimensional free energies as a function of the dihedrals *ϕ* (**E**–**H**) and *ψ* (**I**–**L**), calculated directly from the bias potentials. The same analysis using a dataset with systematic errors is reported in Fig. S7.

Here, we measured the accuracy of the M&M ensembles based on the monodimensional free energies 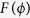 and 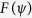 as a function of the two backbone dihedrals of alanine dipeptide. However, the full two-dimensional free-energy surface 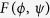 can be obtained from a M&M simulation by standard umbrella-sampling reweighting (Fig. 4), without the need of the more complicated WHAM^26^ approach. Furthemore, more advanced techniques developed for WTMetaD^27^,^28^ should be easily adapted to PBMetaD in order to account more accurately for the time-dependency of the bias potential.

## Discussion

M&M is a modelling approach that combines metainference with metadynamics to model heterogeneous systems by integrating prior knowledge of the system with noisy experimental data. These two methods address very distinct problems, as metainference deals with the problem of accounting for statistical and systematic errors in experimental data collected on heterogeneous systems, while metadynamics deals with the problem of sampling the conformational space efficiently. We benchmarked M&M on the alanine dipeptide in vacuo and demonstrated that sampling is accelerated by the use of the PBMetaD version of metadynamics and that the accuracy of the reconstructed ensemble improves upon adding new experimental data, even if affected by systematic errors. Furthermore, we showed that the noise model that accounts for the presence of outliers is a computationally convenient and accurate alternative to the Gaussian model with one uncertainty parameter per data point.

The M&M method offers the possibility of simulating complex systems with advanced sampling techniques while taking into account all possible sources of errors. However, the application of M&M to more complex biological system will present certain challenges. First, as the complexity of system increases, a greater number of slow degrees of freedom will need to be accelerated and thus included in the set of biased CVs. One possibility will be to devise CVs inspired by different dimensionality reductions tecniques^17^,^29^,^30^ that can efficiently capture the slow modes of complex systems, or to rely on automatic procedures to select the relevant degrees of freedom of a process^31^. In all cases, a major advantage of PBMetaD compared to standard MetaD and to other CV-based enhanced sampling techniques^12^ is that PBMetaD is designed to be used with a large number of CVs, as the high barriers that characterize multi-dimensional free-energy profiles are crossed in a computationally efficient way by simultaneuosly applying multiple parallel bias potentials in low dimensionality. Second, the computational cost required to run MD, and thus M&M, simulations will increase with the size of the system. In this respect, one could be tempted to allocate part of the computational resources available to parallelize the simulation of each replica needed to represent the metainference ensemble. However, since typically the scale in perfomances of MD codes with the number of CPUs used is sub-linear while the sampling efficiency of the multiple-walkers scheme used in M&M scales linearly with the number of replicas, one should dedicate the computational resources available to increase the dimension of the metainference ensemble, rather than to parallelize the simulation of individual replicas. In doing so, one has also the additional advantage of reducing the error in calculating average quantities in the replicas ensemble (Eq. 7).

Finally, an important result presented in the original metainference paper^2^, and naturally extendable to M&M simulations, is that the accuracy of the reconstructed ensemble increases upon adding new experimental data, even when the quality of the prior information is poor. Therefore, to use M&M to model complex biological systems, such as large macromolecular assemblies for which considerable amounts of experimental data are available, a less accurate prior information could be used. Instead of an all-atom explicit-solvent representation of the system, one could rely on an implicit description of the solvent degrees of freedom^32^, or on more coarse-grained potentials, such as MARTINI^33^ or OPEP^34^,^35^. We expect that the poorer quality of this prior information, which guarantees a more computationally efficient exploration of the configurational space, will be compensated in M&M by the introduction of a large amount of data.

We anticipate that the ability of M&M to deal with a wide variety of sources of errors and with heterogeneous systems will make it particularly useful in integrative structural biology^36^, which is based on the combination of different methods, including X-ray crystallography, Förster resonance energy transfer spectroscopy, nuclear magnetic resonance, chemical and cysteine cross-linking, yeast two-hybrid, small-angle X-ray scattering, electron microscopy, and has allowed hybrid models of systems of pivotal biological importance to be determined the last few years^37^,^38^,^39^,^40^,^41^,^42^,^43^,^44^,^45^,^46^,^47^,^48^,^49^,^50^,^51^. To facilitate its use, M&M is implemented in the development version of the PLUMED package and will be available in a future stable release. Implementation in other packages for integrative structure determination^52^,^53^ is also possible.

## Methods

In order to improve the clarity of the presentation reported in the original metainference paper, here we present an extended derivation of the metainference equation in the case of a single experimental data point *d*. This derivation is based on Bayes theorem and on the properties of conditionally independent variables, which are briefly revised in the Supplementary Information (SI). The generalization of this equation to a set of *N*_*d*_ independent experimental data points **D** = [*d*_*i*_] (Eq. 1) is straightforward. In the case of a single data point *d*, the metainference posterior probability of the ensemble of models is


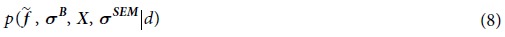


where 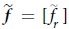 are the averages of the forward model over an infinite number of replicas; 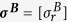 are the uncertainty parameters that describes random and systematic errors in the experimental data as well as errors in the forward model; ***X*** = [*X*_*r*_] are the coordinates of the system; 
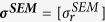
 are the standard errors of the mean associated with the calculation of ensemble averages using few replicas.

We first recognize that 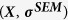 and *d* are conditionally independent given 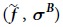



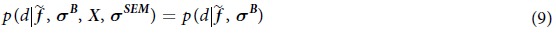


Therefore, we can write the posterior probability as:


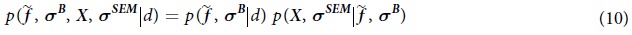


Also 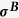 and 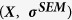 are conditionally independent given 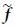



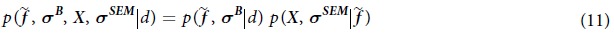


Now we apply Bayes theorem to 
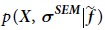
 and we obtain


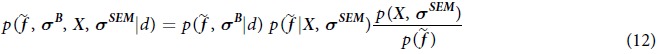


At this point we observe that

(1) 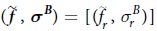 are conditionally independent given *d*


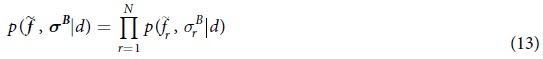


(2) 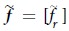 are conditionally independent given 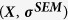



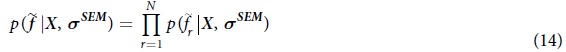



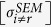
 and 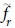 are conditionally independent given 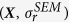



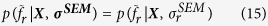



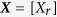
, 
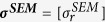
, and 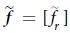 are sets of *a priori* independent variables.

Given the four considerations above, we can rewrite Eq. 12 as


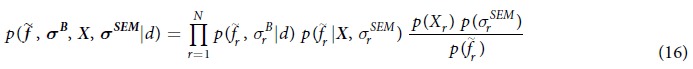


Finally, we apply Bayes theorem to 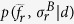 and recognize that 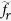 and 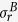 are *a priori* independent


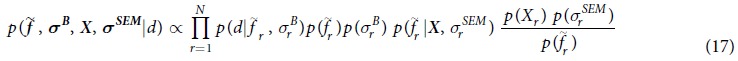


The priors on 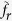 cancel out and we can write the metainference posterior for a single data point *d* as


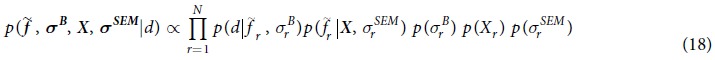


### Gaussian noise model

We can further simplify Eq. 1 in the case of Gaussian noise 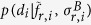 with one uncertainty parameter 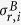 per replica *r* and experimental data point *i*. In this situation 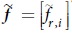 can be marginalized (SI) and the metainference energy function becomes


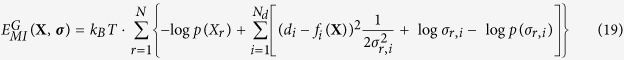


where the effective uncertainty 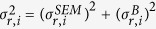 encodes all sources of errors: the statistical errors due to the use of a finite number of replicas, experimental and systematic errors, and errors in the forward model. In the absence of data and forward model errors (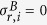), our approach reduces to the replica-averaged MaxEnt modelling, in which a harmonic restraint couples the replica-averaged observable to the experimental data. The intensity of the restraint scales with the number of replicas as *N*^2^, i.e. more than linearly, as required by the MaxEnt principle^54^. In presence of errors (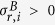), the intensity scales as *N* and it is modulated by the data uncertainty 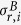. This latter scaling law has also been found in the approach recently proposed by Hummer and Kofinger^55^, although their distribution of the uncertainty parameter is different from the one presented here. Finally, when the experimental data are not ensemble averages (
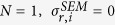
), we recover the standard Bayesian modelling.

### Outliers noise model

To reduce the number of parameters that need to be sampled, the prior on the effective uncertainty 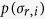 can be modeled using a unimodal distribution centered on a typical dataset uncertainty 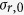 and with a long tail to tolerate outliers^56^. With this choice, each 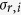 can be marginalized (SI) and the resulting metainference energy function is


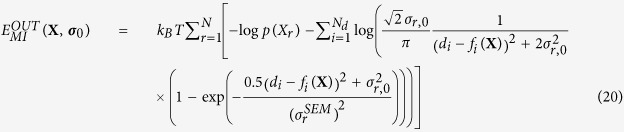


Additional priors 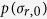 can then be added to model prior knowledge of the typical dataset uncertainty. Here we introduced for the Gaussian and outliers model of Eqs 19 and 20, respectively, a Jeffrey’s prior 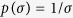; other priors can be used, provided that they make the resulting posterior normalizable.

## Additional Information

**How to cite this article**: Bonomi, M. *et al*. Metadynamic metainference: Enhanced sampling of the metainference ensemble using metadynamics. *Sci. Rep.* **6**, 31232; doi: 10.1038/srep31232 (2016).

## Supplementary Material

Supplementary Information
