# Supplementary material for: Metadynamic metainference: Enhanced sampling of the metainference ensemble using metadynamics
Source: Sci Rep. 2016 Aug 26;6:31232. doi: 10.1038/srep31232 (PMC4999896; doi:10.1038/srep31232)
Supplement: Supplementary Information [file srep31232-s1.pdf]

**SUPPLEMENTARY INFORMATION**

# Metainference Metadynamics

Massimiliano Bonomi[1,*], Carlo Camilloni[1,2] and Michele Vendruscolo[1,*]

[1]*Department of Chemistry, University of Cambridge, Lensfield Road, Cambridge CB2 1EW, UK*

[2]*Department of Chemistry and Institute for Advanced Study, Technische Universität München, Lichtenbergstrasse 4, D-85747 Garching, Germany*

[*]To whom correspondence should be addressed: mb2006@cam.ac.uk; mv245@cam.ac.uk

## Details of the metainference equations

*Properties of conditionally independent variables*. In the derivation of the metainference equations, we make large use of the properties of conditionally independent variables. Here we revise some basic relations. The variables $A$ and $C$ are conditionally independent given $B$ if

$$P(A, C|B) = P(A|B)\, P(C|B) \quad (S1)$$

or equivalently:

$$P(C|A, B) = P(C|B) \quad (S2)$$

Here we demonstate that in this situation the following relation holds:

$$P(A, B|C) = P(B|C)\, P(A|B) \quad (S3)$$

We start by applying Bayes theorem to $P(A, B|C)$:

$$P(A, B|C) = \frac{P(C|A, B)\, P(A, B)}{P(C)} \quad (S4)$$

Now we use the conditionally independence of $A$ and $C$ given $B$:

$$P(A, B|C) = \frac{P(C|B)\, P(A, B)}{P(C)} \quad (S5)$$

and the definition of conditional probability:

$$P(A, B) = P(A|B)\, P(B) \quad (S6)$$

to write:

$$P(A, B|C) = \frac{P(C|B)\, P(A|B)\, P(B)}{P(C)} \quad (S7)$$

If now we apply Bayes theorem to $P(C|B)$, we obtain

$$P(A, B|C) = \frac{P(B|C)\, P(C)}{P(B)} \cdot \frac{P(A|B)\, P(B)}{P(C)} \quad (S8)$$

which leads to Eq. S3:

$$P(A, B|C) = P(B|C)\, P(A|B) \quad (S9)$$

*Gaussian noise model*. Let us consider the metainference posterior for a single data point $d$ in Eq. 18 of the main text:

$$p\left(\tilde{\boldsymbol{f}}, \boldsymbol{\sigma^{B}}, \boldsymbol{X}, \boldsymbol{\sigma^{SEM}} \middle| d\right) \propto \prod_{r=1}^{N} p\left(d|\tilde{f}_r, \sigma_r^B\right)\ p\left(\tilde{f}_r|\boldsymbol{X}, \sigma_r^{SEM}\right) p(\sigma_r^B)\, p(X_r)\, p(\sigma_r^{SEM}) \quad (S10)$$

We can further simplify this expression in the case of Gaussian noise:

$$p\left(d|\tilde{f}_r, \sigma_r^B\right) = \frac{1}{\sqrt{2\pi}\sigma_r^B} \cdot \exp\left[-\frac{\left(d-\tilde{f}_r\right)^2}{2(\sigma_r^B)^2}\right] \quad (S11)$$

In this case, we can write

$$p\left(d|\tilde{f}_r, \sigma_r^B\right) p\left(\tilde{f}_r|\boldsymbol{X}, \sigma_r^{SEM}\right) = \frac{1}{\sqrt{2\pi}\sigma_r^B} \cdot \exp\left[-\frac{\left(d-\tilde{f}_r\right)^2}{2(\sigma_r^B)^2}\right] \cdot \frac{1}{\sqrt{2\pi}\sigma_r^{SEM}} \cdot \exp\left[-\frac{\left(\tilde{f}_r - f(\boldsymbol{X})\right)^2}{2(\sigma_r^{SEM})^2}\right] \quad (S12)$$

The product of the two Gaussian probability density functions (PDFs) is a scaled Gaussian PDF

$$p\left(d|\tilde{f}_r, \sigma_r^B\right) p\left(\tilde{f}_r|\boldsymbol{X}, \sigma_r^{SEM}\right) = \frac{S}{\sqrt{2\pi}\ \bar{\sigma}_r} \cdot \exp\left[-\frac{\left(\tilde{f}_r - \bar{f}\right)^2}{2\bar{\sigma}_r{}^2}\right] \qquad (S13)$$

where

$$\bar{\sigma}_r = \sqrt{\frac{\left(\sigma_r^{SEM}\right)^2 \left(\sigma_r^B\right)^2}{\left(\sigma_r^{SEM}\right)^2 + \left(\sigma_r^B\right)^2}} \quad \text{and} \quad \bar{f} = \frac{d\left(\sigma_r^{SEM}\right)^2 + f(\boldsymbol{X})\left(\sigma_r^B\right)^2}{\left(\sigma_r^{SEM}\right)^2 + \left(\sigma_r^B\right)^2}$$

The scaling factor is itself a Gaussian PDF

$$S = \frac{1}{\sqrt{2\pi\ ((\sigma_r^{SEM})^2 + (\sigma_r^B)^2)}} \exp\left[-\frac{\left(d - f(\boldsymbol{X})\right)^2}{2((\sigma_r^{SEM})^2 + (\sigma_r^B)^2)}\right] \quad (S14)$$

Since typically we are not interested in determining $\tilde{f}_r$, we can marginalize it from Eq. S10 after inserting Eq. S13 and obtain:

$$p(\boldsymbol{X}, \boldsymbol{\sigma}|d) \propto \prod_{r=1}^{N} \int d\tilde{f}_r \, p\left(d|\tilde{f}_r, \sigma_r^B\right) \; p\left(\tilde{f}_r|\boldsymbol{X}, \sigma_r^{SEM}\right) p(\sigma_r^B) \, p(X_r) \, p(\sigma_r^{SEM}) \quad (S15)$$

which leads to:

$$p(\boldsymbol{X}, \boldsymbol{\sigma}|d) \propto \prod_{r=1}^{N} \frac{1}{\sqrt{2\pi}\sigma_r} \exp\left[-\frac{\left(d - f(\boldsymbol{X})\right)^2}{2\sigma_r^2}\right] p(X_r) \, p(\sigma_r) \quad (S16)$$

where the effective uncertainty parameters $\sigma_r = \sqrt{(\sigma_r^{SEM})^2 + (\sigma_r^B)^2}$ encodes all sources of error.

Similary, the posterior for multiple independent data points $\mathbf{D} = [d_i]$ in Eq. 1 of the main text:

$$p\left(\tilde{\boldsymbol{f}}, \boldsymbol{\sigma}^{\boldsymbol{B}}, \boldsymbol{X}, \boldsymbol{\sigma}^{\boldsymbol{SEM}}|\mathbf{D}\right) = \prod_{r=1}^{N} p(X_r) \prod_{i=1}^{N_d} p\left(d_i\middle|\tilde{f}_{r,i}, \sigma_{r,i}^B\right) \cdot p\left(\tilde{f}_{r,i}\middle|\mathbf{X}, \sigma_{r,i}^{SEM}\right) \cdot p(\sigma_{r,i}^{SEM}) \cdot p(\sigma_{r,i}^B) \quad (S17)$$

can be simplified in case of Gaussian noise on all data points:

$$p(\boldsymbol{X}, \boldsymbol{\sigma}|\boldsymbol{D}) \propto \prod_{r=1}^{N} p(X_r) \prod_{i=1}^{N_d} \frac{1}{\sqrt{2\pi}\sigma_{r,i}} \exp\left[-\frac{(d_i - f_i(\boldsymbol{X}))^2}{2\sigma_{r,i}^2}\right] \; p\left(\sigma_{r,i}\right) \quad (S18)$$

which leads to the metainference energy function in Eq. 19 of the main text:

$$E_{MI}^G(\mathbf{X}, \boldsymbol{\sigma}) = k_B T \cdot \sum_{r=1}^{N} \left\{ -\log p(X_r) + \sum_{i=1}^{N_d} \left[ \left(d_i - f_i(\mathbf{X})\right)^2 \frac{1}{2\sigma_{r,i}^2} + \log \sigma_{r,i} - \log p\left(\sigma_{r,i}\right) \right] \right\} \quad (S19)$$

*Outliers noise model*. To reduce the number of parameters that need to be sampled in the case of multiple experimental data points, one can model the distribution of the errors around a typical dataset error and marginalize the error parameters for the individual data points. For example, a dataset can be defined as a set of chemical shifts or RDCs on a given nucleus. In this cases, it is reasonable to assume that the level of error of the individual data points in the dataset is homogenoues, except for the presence of few outliers. Let us consider for example the case of Gaussian data noise in presence of multiple experimental data points:

$$p(\boldsymbol{X}, \boldsymbol{\sigma}|\boldsymbol{D}) \propto \prod_{r=1}^{N} p(X_r) \prod_{i=1}^{N_d} \frac{1}{\sqrt{2\pi}\sigma_{r,i}} \exp\left[-\frac{(d_i - f_i(\boldsymbol{X}))^2}{2\sigma_{r,i}^2}\right] p(\sigma_{r,i}) \quad (S20)$$

The prior $p(\sigma_{r,i})$ can be modeled using a unimodal distribution peaked around a typical dataset effective uncertainty $\sigma_{r,0}$ and with a long tail to tolerate outliers data points:

$$p(\sigma_{r,i}) = \frac{2\sigma_{r,0}}{\sqrt{\pi}\sigma_{r,i}^2} \exp\left(-\frac{\sigma_{r,0}^2}{\sigma_{r,i}^2}\right) \quad (S21)$$

where $\sigma_{r,0} = \sqrt{(\sigma_r^{SEM})^2 + \left(\sigma_{r,0}^B\right)^2}$, with $\sigma_r^{SEM}$ is the the standard error of the mean for all data points in the dataset and $\sigma_{r,0}^B$ is the typical data uncertainty of the dataset. We can thus marginalize $\sigma_{r,i}$ by integrating over all its possible values, given that all the data uncertainties $\sigma_{r,i}^B$ range from 0 to infinity:

$$p(\boldsymbol{X}, \boldsymbol{\sigma_0}|\boldsymbol{D}) \propto \prod_{r=1}^{N} p(X_r) \prod_{i=1}^{N_d} \int_{\sigma_r^{SEM}}^{+\infty} d\sigma_{r,i} \, \frac{\sqrt{2}\,\sigma_{r,0}}{\pi\,\sigma_{r,i}^3} \exp\left[-\frac{0.5\,(d_i - f_i(\boldsymbol{X}))^2 + \sigma_{r,0}^2}{\sigma_{r,i}^2}\right] \quad (S22)$$

which leads to:

$$p(\boldsymbol{X}, \boldsymbol{\sigma_0} | \boldsymbol{D}) \propto \prod_{r=1}^{N} p(X_r) \prod_{i=1}^{N_d} \frac{\sqrt{2}\, \sigma_{r,0}}{\pi} \frac{1}{(d_i - f_i(\boldsymbol{X}))^2 + 2\sigma_{r,0}^2} \left\{1 - \exp\left[-\frac{0.5\, (d_i - f_i(\boldsymbol{X}))^2 + \sigma_{r,0}^2}{(\sigma_r^{SEM})^2}\right]\right\} \quad (S23)$$

and to the metainference energy function in Eq. 20 of the main text:

$$E_{MI}^{OUT}(\mathbf{X}, \boldsymbol{\sigma_0}) = k_B T \sum_{r=1}^{N} \left[ -\log p(X_r) - \sum_{i=1}^{N_d} \log\left( \frac{\sqrt{2}\sigma_{r,0}}{\pi} \frac{1}{\left(d_i - f_i(\mathbf{X})\right)^2 + 2\sigma_{r,0}^2} \left( 1 - \exp\left( - \frac{0.5\left(d_i - f_i(\mathbf{X})\right)^2 + \sigma_{r,0}^2}{(\sigma_r^{SEM})^2} \right) \right) \right) \right] \quad (S24)$$

**Computational details**

*Data generation***.** The goal of this step is to calculate the average of the 36 distances between all the (non-bonded) heavy atoms of the alanine dipeptide in the ensemble generated by a modified version of the AMBER99SB-ILDN[1] force field, in which the local minima $C_{ax}$ is shifted down in free energy by 15 kJ/mol. We defined the free energy of this ensemble as a function of the backbone dihedrals $\Phi$ and $\Psi$ as

$$\tilde{F}(\Phi,\Psi) = F(\Phi,\Psi) - 15.0 \cdot \exp[-(\Phi - 1.0)^2] \qquad (S25)$$

where $F(\Phi,\Psi)$ is the free energy calculated with AMBER99SB-ILDN. In Fig. 4A,B we report $F(\Phi,\Psi)$ and $\tilde{F}(\Phi,\Psi)$, respectively. The monodimensional projections $\tilde{F}(\Phi)$ and $\tilde{F}(\Psi)$ are shown as black lines in Fig. 2A and Fig. 2D, respectively; $F(\Phi)$ and $F(\Psi)$ are shown as red lines in Fig. 2A and Fig. 2D, respectively.

The procedure used to calculate the averages in the ensemble given by Eq. S25 consisted of four steps:

1) We run a 200 ns-long well-tempered metadynamics[2] (WTMetaD) calculation using the AMBER99SB-ILDN force field and the two dihedrals $\Phi$ and $\Psi$ as collective variables (CVs). A Gaussian width of 0.35 rad was used for both CVs, along with an initial Gaussian height of 1.2 kJ/mol, a bias factor of 8, and a deposition stride of 1 ps.

2) We run a 200 ns-long molecular dynamics simulation using the AMBER99SB-ILDN force field and the static WTMetaD bias potential $V_G(\Phi,\Psi)$ obtained at the end of step 1. Under the effect of the bias, the system explored all the relevant regions of the Ramachandran[3] plot. In this simulation, configurations were saved every 0.2 ps, for a total of $10^6$ frames.

3) We back-calculated using the *driver* utility of PLUMED[4] all the 36 distances from the trajectory obtained at step 2. We used these distances to calculate the averages in the ensemble defined by Eq. S25. In order to do so, we had to reweight each frame to eliminate the effect of the static WTMetaD bias potential, which ensured ergodicity, and to add the offset in the free energy of the $C_{ax}$ local minimum. Therefore, in the calculation of the average distances from the trajectory generated at step 2, each frame $X_i$ was assigned the following weight[5]

$$w(X_i) = \exp\{[V_G(\Phi_i, \Psi_i) + 15.0 \cdot \exp(-(\Phi_i - 1.0)^2)]/k_B T\} \quad (S26)$$

where $k_B$ is the Boltzman constant and $T$ is the temperature of the system. The final averages are reported in Tab. S1 (third column) along with the average distances calculated separately in the region of $C_{7\mathrm{eq}}$ ($\Phi < 0$, fourth column) and $C_{\mathrm{ax}}$ ($\Phi > 0$, fifth column). The average distances in the AMBER99SB-ILDN ensemble are reported in Tab. S1, sixth column.

4) To introduce systematic errors in the pure (synthetic) data calculated at step 3, we added a random offset in the range from 0.2 to 0.3 nm to 20% of the final averages reported in Table S1.

*Metainference Metadynamics (M&M) simulations details*. All simulations were carried out using GROMACS[6] 4.6.7 equipped with PLUMED (development branch). The system was simulated in vacuo, without cutoffs on electrostatic and van der Waals interactions. A time step of 2 fs was used. Bonds were constrained using the LINCS algorithm[7]. The constant temperature of 300 K was enforced by the Bussi-Donadio-Parrinello thermostat[8], with coupling constant equal to 0.1 ps. All simulations were carried out for an aggregated simulation time (simulation time per replica multiplied by the number of replicas) of 120 ns. For the metainference[9] part, uncertainties were sampled by a MC scheme in the range from 0.00001 to 10.0, using a maximum step for MC move equal to 0.1. $\sigma^{SEM}$ was fixed at $\sigma^{SEM} = 0.12/\sqrt{N}$, where $N$ is the number of replicas used.

For the parallel bias metadynamics[10] (PBMetaD) part, we used as CVs the 4 dihedrals $\phi$, $\psi$, $\theta$, and $\zeta$ defined as follows:

- $\phi$: atoms C–N–Cα–C
- $\psi$: atoms N–Cα–C–N
- $\theta$: atoms O–C–N–Cα
- $\zeta$: atoms Cα–C–N–H

We used Gaussian widths and bias factors equal to 0.35 rad and 8 for all CVs, respectively, an initial Gaussian height of 1.2 kJ/mol, and a deposition stride of 1 ps. Replicas shared the mono-dimensional bias potentials as in the multiple-walkers technique[11].

For the bias exchange metadynamics[12] part (BEM), we used the 4 dihedrals defined above as CVs and the same Gaussian parameters as in PBMetaD. Exchanges were attempted every 1000 MD steps. Each metainference replica used one dihedral as CV. Since we utilized a total of 8 replicas and 4 CVs, each CV was biased by 2 different replicas.

*Metainference simulations details*. All the simulations were performed using the same parameters of the M&M runs described above.

*Analysis*. To quantify the accuracy of M&M, we measured the root mean square deviations (RMSDs) of $\tilde{F}(\Phi)$ and $\tilde{F}(\Psi)$ from the estimates $\tilde{F}_{M\&M}(\Phi)$ and $\tilde{F}_{M\&M}(\Psi)$ obtained from the PBMetaD bias potential at the end of the simulation

$$RMSD(\tilde{F}, \tilde{F}_{M\&M}) = \sqrt{\frac{1}{\Omega}\int_{\Omega} dS \left[\left(\tilde{F}(S) - \langle\tilde{F}(S)\rangle\right) - \left(\tilde{F}_{M\&M}(S) - \langle\tilde{F}_{M\&M}(S)\rangle\right)\right]^2} \quad (S27)$$

where $S$ is either $\Phi$ or $\Psi$, $\langle\tilde{F}(S)\rangle$ and $\langle\tilde{F}_{M\&M}(S)\rangle$ are the reference and estimated free energies averaged over the region $\Omega$. Since sampling in M&M is limited to relevant regions of the CV space by the PBMetaD temperature parameter $\Delta T$, $\Omega$ were defined as the regions within 20 kJ/mol of the global minima of the references $\tilde{F}(S)$.

To calculate the full two-dimensional free energy $\tilde{F}_{M\&M}(\Phi, \Psi)$, we used a simple reweighting procedure. Since the PBMetaD bias potential $V_{PB}(\Phi, \Psi, t)$ becomes quasi-static in the long-time limit, we discarded the initial transient (20% of the entire run) and considered the bias potential as static for the remaining of the simulation. We then used the standard Torrie-Valleau umbrella sampling reweighting[5] to recover the unbiased probability distribution by assigning to each conformation the weight $w(\Phi, \Psi) \propto e^{+\frac{V_{PB}(\Phi,\Psi,\bar{t})}{k_B T}}$, where $V_{PB}(\Phi, \Psi, \bar{t})$ is the PBMetaD bias potential at the end of the simulation.

## References


1 Lindorff-Larsen, K. *et al*. Improved side-chain torsion potentials for the Amber ff99SB protein force field. *Proteins* **78**, 1950-1958, (2010).

2 Barducci, A., Bussi, G. & Parrinello, M. Well-tempered metadynamics: A smoothly converging and tunable free-energy method. *Phys. Rev. Lett.* **100**, (2008).

3 Ramachandran, G. N., Ramakrishnan, C. & Sasisekharan, V. Stereochemistry of Polypeptide Chain Configurations. *J. Mol. Biol.* **7**, 95-&, (1963).

4 Tribello, G. A., Bonomi, M., Branduardi, D., Camilloni, C. & Bussi, G. PLUMED 2: New feathers for an old bird. *Comp. Phys. Comm.* **185**, 604-613, (2014).

5 Torrie, G. M. & Valleau, J. P. Non-Physical Sampling Distributions in Monte-Carlo Free-Energy Estimation - Umbrella Sampling. *J. Comput. Phys.* **23**, 187-199, (1977).

6 Hess, B., Kutzner, C., van der Spoel, D. & Lindahl, E. GROMACS 4: Algorithms for highly efficient, load-balanced, and scalable molecular simulation. *J. Chem. Theory Comput.* **4**, 435-447, (2008).

7 Hess, B., Bekker, H., Berendsen, H. J. C. & Fraaije, J. G. E. M. LINCS: A linear constraint solver for molecular simulations. *J. Comput. Chem.* **18**, 1463-1472, (1997).

8 Bussi, G., Donadio, D. & Parrinello, M. Canonical sampling through velocity rescaling. *J. Chem. Phys.* **126**, (2007).

9 Bonomi, M., Camilloni, C., Cavalli, A. & Vendruscolo, M. Metainference: a Bayesian inference method for heterogeneous systems. *Sci. Adv.* **2**, e1501177, (2016).

10 Pfaendtner, J. & Bonomi, M. Efficient sampling of high-dimensional free-energy landscapes with Parallel Bias Metadynamics. *J. Chem. Theory Comput.* **11**, 5062-5067, (2015).

11 Raiteri, P., Laio, A., Gervasio, F. L., Micheletti, C. & Parrinello, M. Efficient reconstruction of complex free energy landscapes by multiple walkers metadynamics. *J. Phys. Chem. B* **110**, 3533-3539, (2006).

12 Piana, S. & Laio, A. A bias-exchange approach to protein folding. *J. Phys. Chem. B* **111**, 4553-4559, (2007).

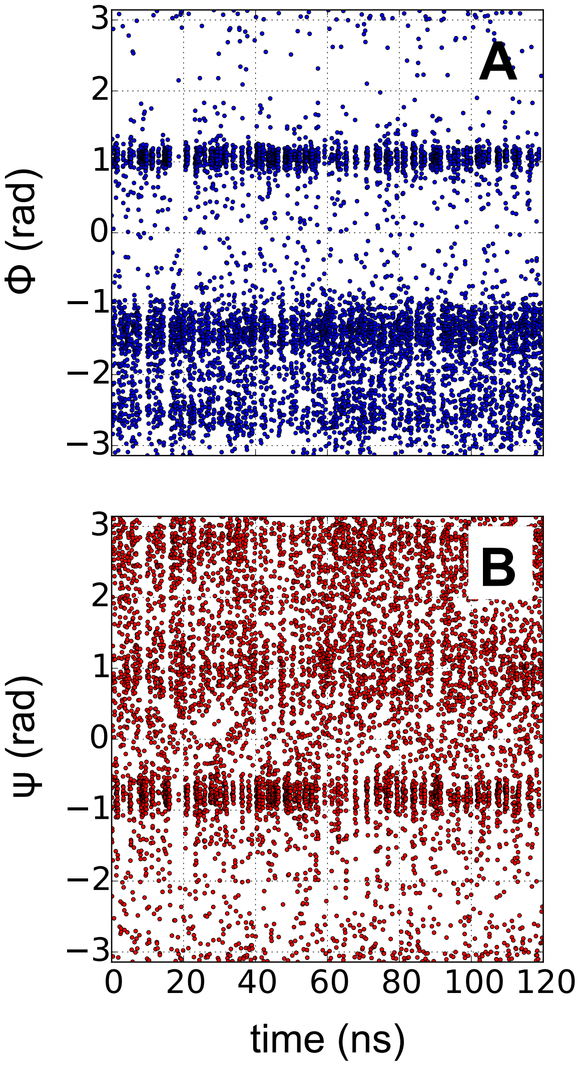


**Figure S1.** Assessment of the ergodicity of the M&M simulations of the alanine dipeptide in vacuo. We visualize the diffusion in the space of the CVs $\Phi$ (A) and $\Psi$ (B) to ensure that all the relevant regions of the Ramachandran plot were visited under the effect of the PBMetaD bias potential. This is a representative example of a M&M simulation using 8 replicas, all the 36 data points available (without addition of systematic errors), and the Gaussian noise model.

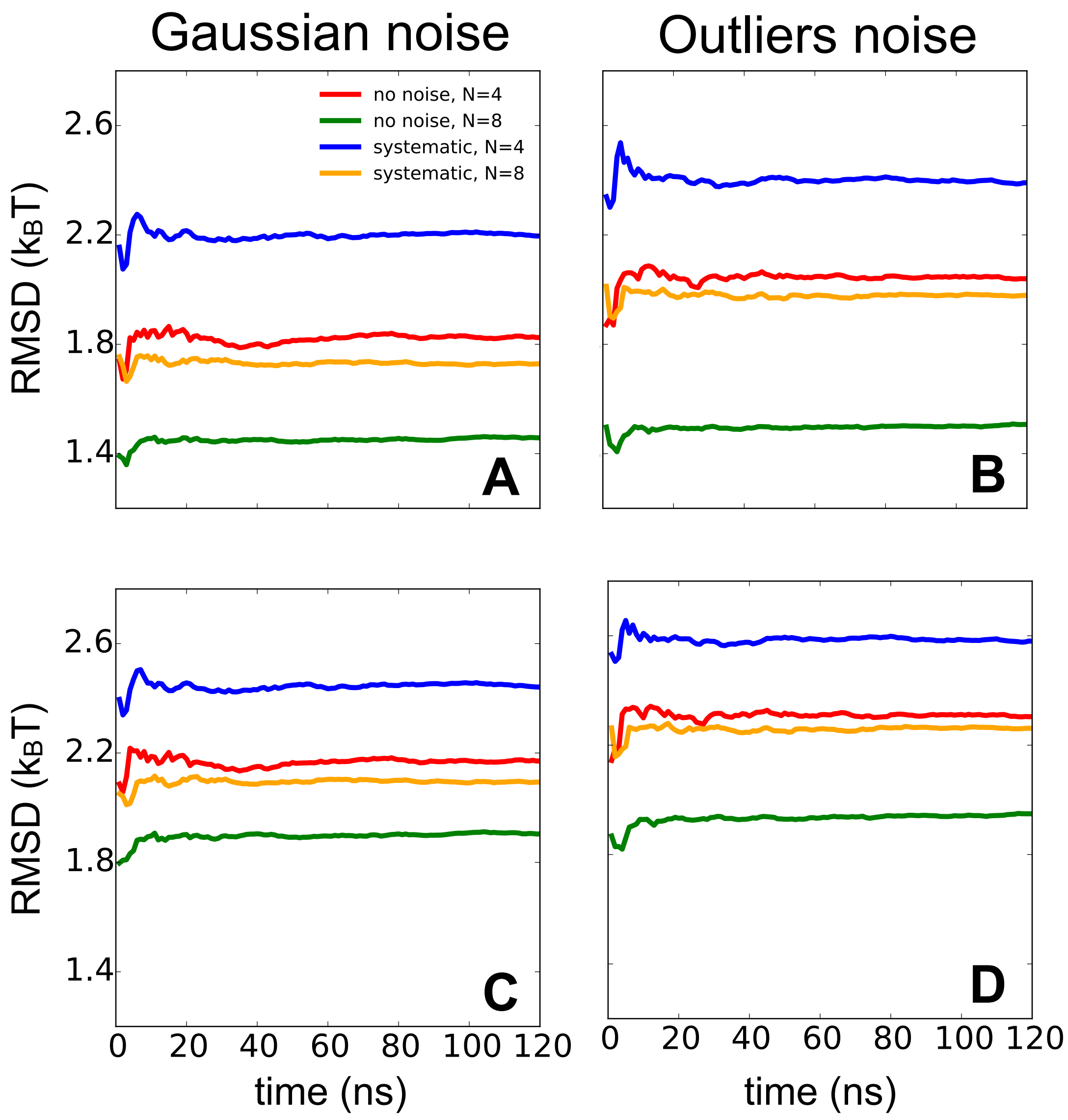


**Figure S2.** Assessment of the convergence of the M&M simulations of the alanine dipeptide in vacuo. We calculated, as a function of simulation time, the RMSD of the estimates $\tilde{F}_{M\&M}(\Phi)$ and $\tilde{F}_{M\&M}(\Psi)$ of the free energies along the dihedrals $\Phi$ (upper panels) and $\Psi$ (lower panels) from the reference profiles $\tilde{F}(\Phi)$ and $\tilde{F}(\Psi)$. Here we report the RMSD averaged over 100 independent simulations, in the case of Gaussian (AC) and outliers noise models (BD) as a function of the number of replicas and the level of noise in the data. The number of data points used is 36.

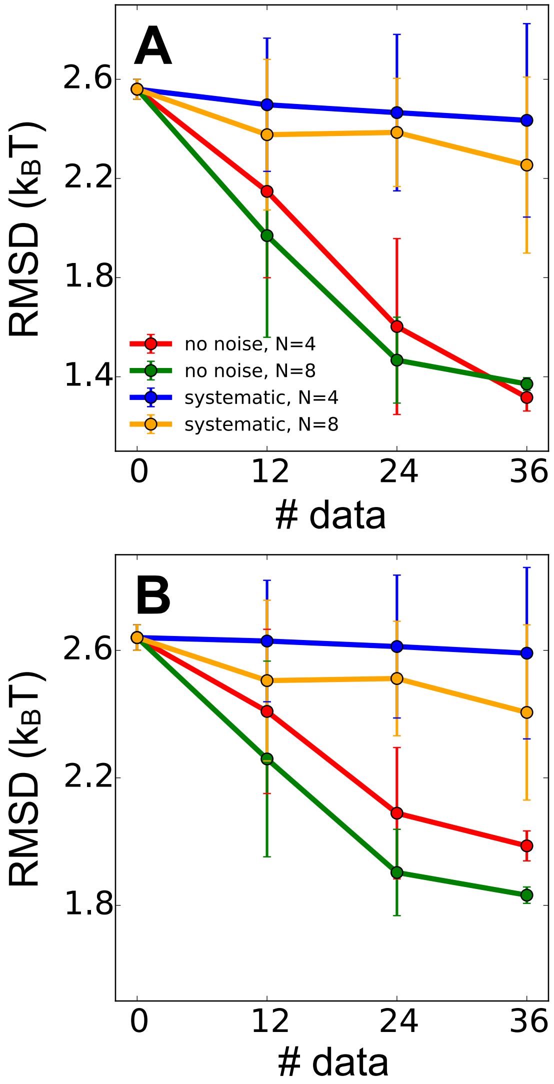


**Figure S3.** Benchmark of the M&M accuracy on the alanine dipeptide in vacuo as a function of the number of data points, the level of noise in the data, and the number of replicas used, using a Gaussian noise model with one uncertainty parameter for all data points.

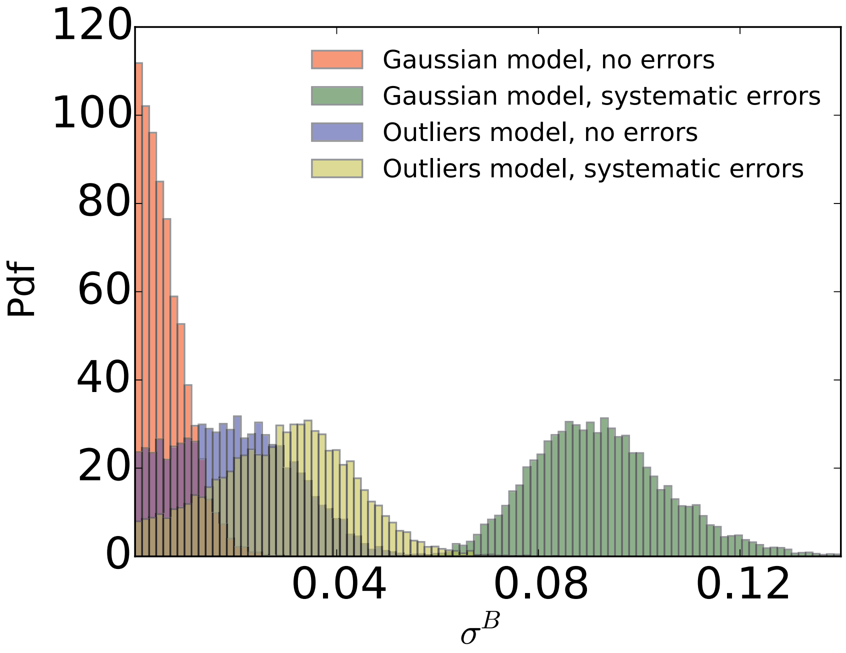


**Figure S4.** Analysis of the uncertainties inferred by M&M. The distributions of the $\sigma^B$ parameter in representative simulations with 8 replicas and all the 36 data points available, using the Gaussian noise model with one uncertainty parameter for all data points, in absence (red) and presence (green) of systematic errors, and the outliers model (Eq. 20), in absence (blue) and presence (yellow) of systematic errors. We used the same dataset with systematic errors for both the Gaussian and the outliers models. In the former case, $\sigma^B$ is the single uncertainty parameter used for all data points; in the latter, $\sigma^B$ is the typical uncertainty of the dataset.

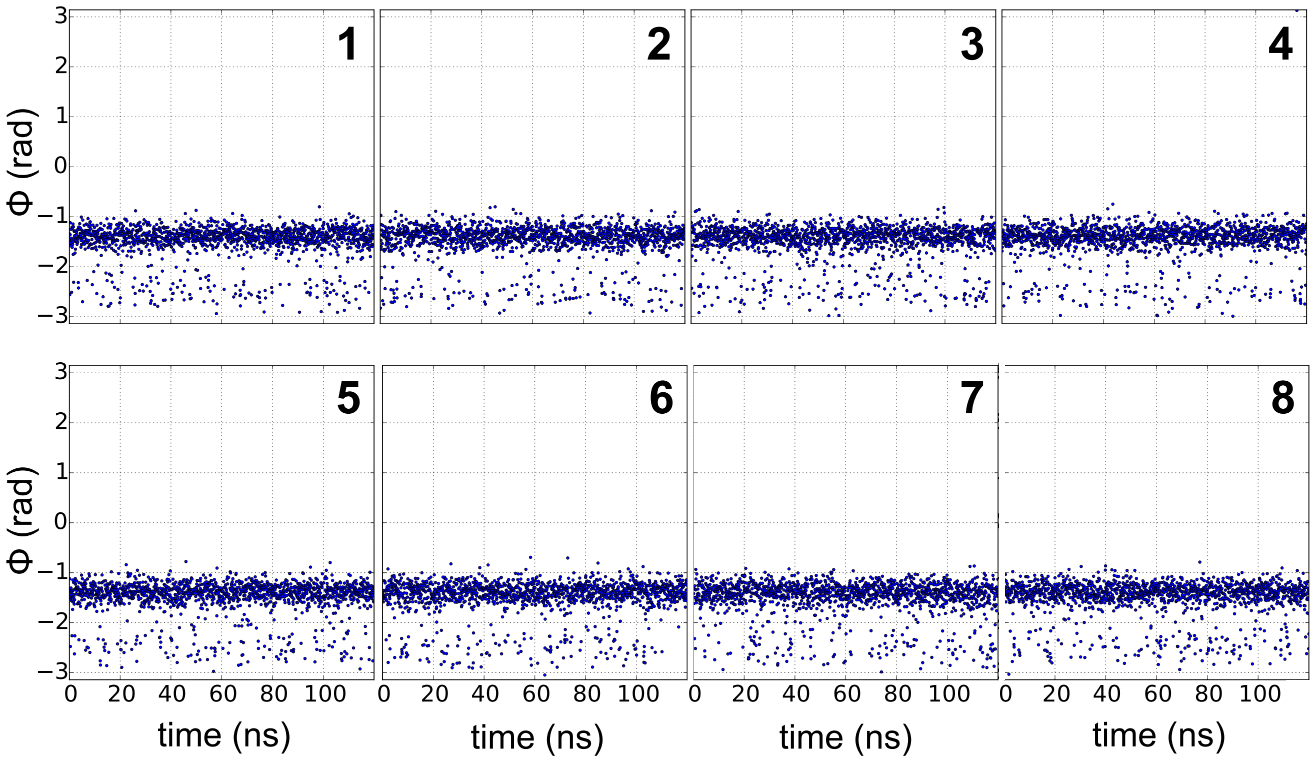


**Figure S5.** Assessment of the non-ergodicity of the Metainference simulations of the alanine dipeptide in vacuo. We visualize the diffusion in the space of the CV $\Phi$ to demonstrate that, in the absence of the PBMetaD bias potential, the system could not explore all the relevant regions of the Ramachandran plot. This is a representative example of a Metainference simulation using 8 replicas, all the 36 data points available (without addition of systematic errors), and the Gaussian noise model. Each panel represent the time evolution of a particular replica. In this run, the local minimum located around $\Phi =$ 1 was never explored.

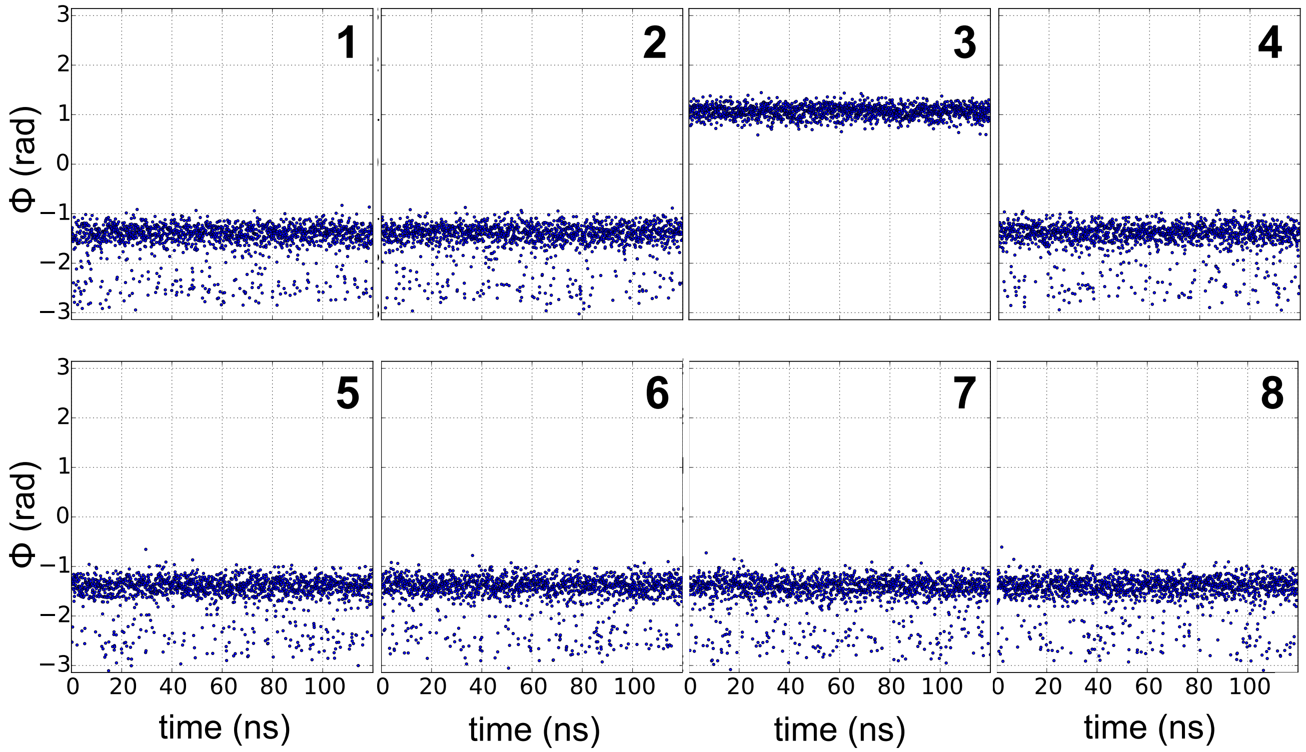


**Figure S6.** Assessment of the non-ergodicity of the Metainference simulations of the alanine dipeptide in vacuo. We visualize the diffusion in the space of the CV $\Phi$ to demonstrate that, in the absence of the PBMetaD bias potential, each replica could not explore all the relevant regions of the Ramachandran plot. This is a representative example of a Metainference simulation using 8 replicas, all the 36 data points available (without addition of systematic errors), and the Gaussian noise model. Each panel represent the time evolution of a particular replica. In this run, replica remained trapped in the starting basin and the results were thus influenced by the initial distribution of replicas.

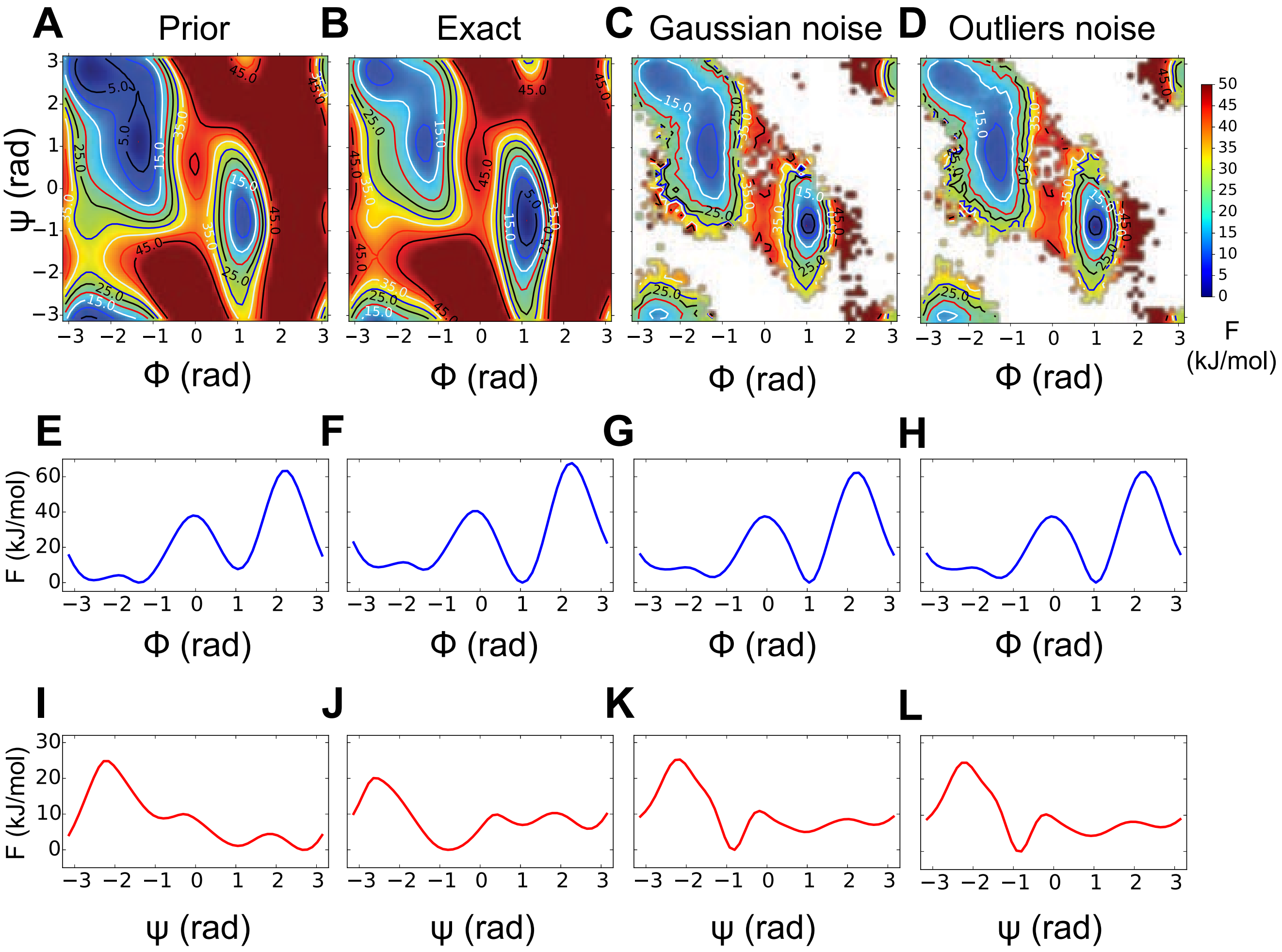


**Figure S7.** Reweighting of M&M simulations. Free energy of alanine dipeptide as a function of the backbone dihedrals obtained with the AMBER99SB-ILDN prior alone (A) and with the correction to lower the free energy of the local minimum $C_{\mathrm{ax}}$ (B). The latter is considered our reference (exact) free energy. Free energy obtained from reweighting a M&M simulation carried out using 64 replicas, all the 36 data points available (with the addition of systematic errors), the Gaussian noise model with one uncertainty parameter for each data point (C) and the outliers noise model with one parameter per dataset (D). The visualization is truncated at 50 kJ/mol from the global minimum. For each case (prior, exact, Gaussian noise, outliers noise), we also reported the monodimensional free energies as a function of the dihedrals $\phi$ (E, F, G, H) and $\psi$ (I, J, K, L), calculated directly from the bias potentials.

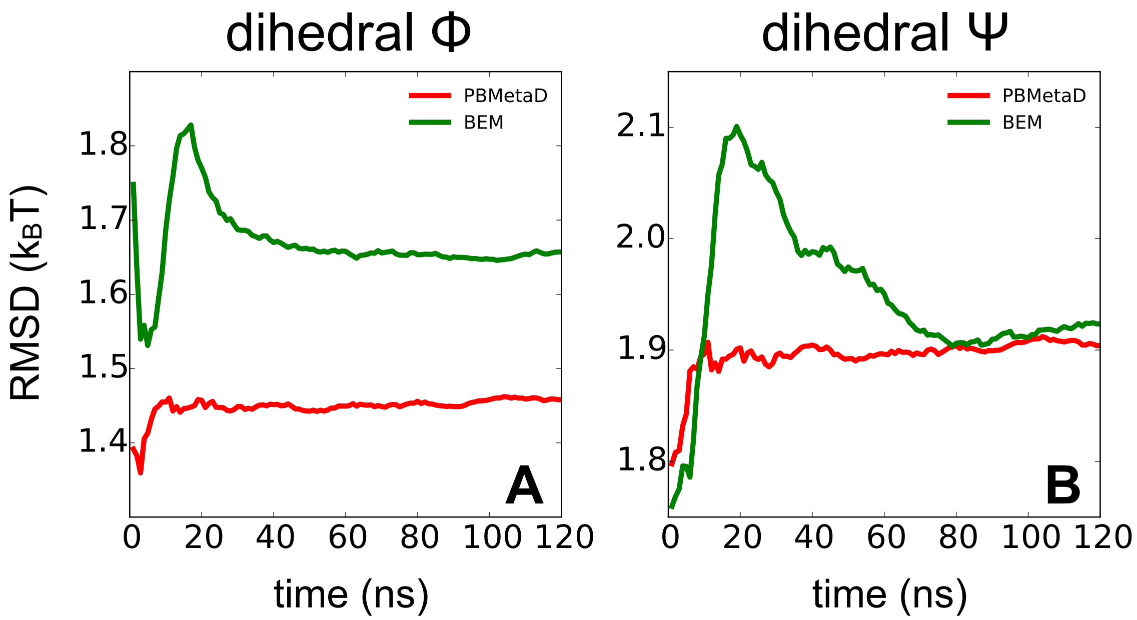


**Figure S8.** Comparison of the efficiency and accuracy of BEM and PBMetaD combined with metainference. We calculated, as a function of simulation time, the error in the estimated free energies along the dihedrals $\Phi$ (A) and $\Psi$ (B) obtained with BEM (green lines) and PBMetaD (red lines). Here we report the RMSD from the reference profiles averaged over 100 independent simulations, in the case of Gaussian noise model with one uncertainty parameter per data point, in absence of systematic errors in the data, using 36 data points and 8 replicas.

| ID 1 | ID 2 | $\langle d \rangle$ | $\langle d \rangle_{C_{7eq}}$ | $\langle d \rangle_{C_{ax}}$ | $\langle d \rangle_{prior}$ |
|---|---|---|---|---|---|
| 2 | 6 | 0.238 | 0.239 | 0.238 | 0.239 |
| 2 | 7 | 0.243 | 0.243 | 0.243 | 0.243 |
| 2 | 9 | 0.382 | 0.381 | 0.383 | 0.381 |
| 2 | 11 | 0.452 | 0.467 | 0.450 | 0.466 |
| 2 | 15 | 0.460 | 0.465 | 0.460 | 0.465 |
| 2 | 16 | 0.559 | 0.512 | 0.565 | 0.513 |
| 2 | 17 | 0.459 | 0.524 | 0.450 | 0.523 |
| 2 | 19 | 0.574 | 0.638 | 0.566 | 0.637 |
| 5 | 9 | 0.250 | 0.246 | 0.251 | 0.247 |
| 5 | 11 | 0.320 | 0.349 | 0.316 | 0.348 |
| 5 | 15 | 0.325 | 0.342 | 0.322 | 0.341 |
| 5 | 16 | 0.432 | 0.411 | 0.435 | 0.411 |
| 5 | 17 | 0.326 | 0.396 | 0.316 | 0.396 |
| 5 | 19 | 0.453 | 0.523 | 0.444 | 0.522 |
| 6 | 7 | 0.226 | 0.225 | 0.227 | 0.225 |
| 6 | 9 | 0.292 | 0.284 | 0.293 | 0.284 |
| 6 | 11 | 0.336 | 0.391 | 0.328 | 0.389 |
| 6 | 15 | 0.333 | 0.372 | 0.327 | 0.372 |
| 6 | 16 | 0.448 | 0.460 | 0.446 | 0.460 |
| 6 | 17 | 0.296 | 0.396 | 0.282 | 0.395 |
| 6 | 19 | 0.411 | 0.521 | 0.395 | 0.519 |
| 7 | 11 | 0.243 | 0.241 | 0.243 | 0.241 |
| 7 | 15 | 0.253 | 0.246 | 0.254 | 0.246 |
| 7 | 16 | 0.346 | 0.305 | 0.352 | 0.306 |
| 7 | 17 | 0.305 | 0.332 | 0.301 | 0.332 |
| 7 | 19 | 0.443 | 0.462 | 0.440 | 0.462 |
| 9 | 16 | 0.237 | 0.239 | 0.237 | 0.239 |
| 9 | 17 | 0.246 | 0.244 | 0.247 | 0.244 |
| 9 | 19 | 0.384 | 0.381 | 0.384 | 0.381 |
| 11 | 15 | 0.254 | 0.254 | 0.254 | 0.254 |
| 11 | 16 | 0.324 | 0.306 | 0.327 | 0.307 |
| 11 | 17 | 0.331 | 0.346 | 0.329 | 0.346 |
| 11 | 19 | 0.462 | 0.475 | 0.460 | 0.474 |
| 15 | 19 | 0.247 | 0.247 | 0.247 | 0.247 |
| 16 | 17 | 0.224 | 0.224 | 0.224 | 0.224 |
| 16 | 19 | 0.282 | 0.282 | 0.282 | 0.282 |

**Table S1.** Average distances (in nm) between all pairs of (non-bonded) heavy atoms of alanine dipeptide, calculated in the reference ensemble (third column), only in the regions of the $C_{7eq}$ (fourth column) and $C_{ax}$ (fifth column) local minima, and in the ensemble generated by the AMBER99SB-ILDN prior (sixth column). The atom IDs in the first and second columns correspond to the atom numbers in the PDB file reported below (second column). The metainference score is defined as a function of the atom (average) distances defined here.

```
ATOM      1 1HH3  ACE   1
ATOM      2  CH3  ACE   1
ATOM      3 2HH3  ACE   1
ATOM      4 3HH3  ACE   1
ATOM      5  C    ACE   1
ATOM      6  O    ACE   1
ATOM      7  N    ALA   2
ATOM      8  H    ALA   2
ATOM      9  CA   ALA   2
ATOM     10  HA   ALA   2
ATOM     11  CB   ALA   2
ATOM     12  HB1  ALA   2
ATOM     13  HB2  ALA   2
ATOM     14  HB3  ALA   2
ATOM     15  C    ALA   2
ATOM     16  O    ALA   2
ATOM     17  N    NME   3
ATOM     18  H    NME   3
ATOM     19  CH3  NME   3
ATOM     20 1HH3  NME   3
ATOM     21 2HH3  NME   3
ATOM     22 3HH3  NME   3
```
